# High fat diet accelerates and exacerbates microgliosis and neuronal damage/death in the somatosensory cortex after transient forebrain ischemia in gerbils

**DOI:** 10.1186/s42826-020-00061-1

**Published:** 2020-08-20

**Authors:** Won Joo Seo, Ji Hyeon Ahn, Tae-Kyeong Lee, Bora Kim, Jae-Chul Lee, Joon Ha Park, Yeon Ho Yoo, Myoung Cheol Shin, Jun Hwi Cho, Moo-Ho Won, Yoonsoo Park

**Affiliations:** 1 Department of Emergency Medicine, and Institute of Medical Sciences, Kangwon National University Hospital, School of Medicine, Kangwon National University, Chuncheon, Gangwon 24341 Republic of Korea; 2 Department of Biomedical Science and Research Institute for Bioscience and Biotechnology, Hallym University, Chuncheon, Gangwon 24252 Republic of Korea; 3 Department of Neurobiology, School of Medicine, Kangwon National University, Chuncheon, Gangwon 24341 Republic of Korea; 4 Department of Anatomy, College of Oriental Medicine, Dongguk University-Gyeongju, Gyeongju, Gyeongbuk 38066 Republic of Korea

**Keywords:** Ischemic stroke, Microgliosis, Neuronal death, Obesity, Somatosensory cortex

## Abstract

Obesity has been known as an independent risk factor for stroke. Effects of high-fat diet (HFD)-induced obesity on neuronal damage in the somatosensory cortex of animal models of cerebral ischemia have not been studied yet. In this study, HFD-induced obesity was used to study the impact of obesity on neuronal damage/loss and microgliosis in the somatosensory cortex of a gerbil model of 5-min transient forebrain ischemia. We used gerbils fed normal diet (ND) and HFD and chronologically examined microgliosis (microglial cell activation) by ionized calcium-binding adapter molecule 1 (Iba-1) immunohistochemistry. In addition, we examined neuronal damage or death by using neuronal nuclear protein (NeuN, a neuronal marker) immunohistochemistry and Fluoro-Jade B (F-J B, a marker for neuronal degeneration) histofluorescence staining. We found that ischemia-induced microgliosis in ND-fed gerbils was increased from 2 days post-ischemia; however, ischemia-mediated microgliosis in HFD-fed gerbils increased from 1 day post-ischemia and more accelerated with time than that in the ND-fed gerbils. Ischemia-induced neuronal death/loss in the somatosensory cortex in the ND-fed gerbils was apparently found at 5 days post-ischemia. However, in the HFD-fed gerbils, neuronal death/loss was shown from 2 days post-ischemia and progressively exacerbated at 5 days post-ischemia. Our findings indicate that HFD can evoke earlier microgliosis and more detrimental neuronal death/loss in the somatosensory cortex after transient ischemia than ND evokes.

## Introduction

Obesity caused by high-fat diet (HFD) is one of the most serious public health problems worldwide. Obesity has been known as a major risk factor for brain ischemia [1]. The obese individuals with stroke experiences more complications, longer hospitalization stays, and have worse functional improvement than lean individuals [2–5]. Obesity is also a strong risk factor for the development of hypertension, diabetes and dyslipidemia, which can lead to a negative effect on stroke outcome clinically and in experimental models [6, 7]. Our previous study designed a model of obese gerbils that are fed HFD for 12 weeks [1]. These results showed that HFD-induced obesity exacerbates brain damage in animal models of transient cerebral ischemia in gerbils [1, 6, 8–10]. HFD-induced obesity in rodents results in larger infarcts size and more severe behavioral deficits after ischemia insults [2]. Diet-induced obesity causes cerebral vessel remodeling with increased stiffness, which increases ischemic damage after middle cerebral artery occlusion in mice and rats [11–13]. Another study showed that HFD exacerbates ischemic damage following traumatic brain injury [11]. HFD fed rats and mice for 8–12 weeks profoundly increases cerebral infarction following transient focal cerebral ischemia [1, 8, 9, 14].

Transient ischemic insults in the brain causes neuronal damage and death in specifically vulnerable regions and leads to severe neurologic impairments [15, 16]. Ischemia/reperfusion injury following transient global cerebral ischemia is a major cause of neurologic abnormalities, including seizures, delirium, neurocognitive impairment, etc. [16, 17]. It has been demonstrated that many regions of the brain are selectively vulnerable to transient global cerebral ischemia [18]. The vulnerable regions include the cerebral cortex, striatum and hippocampus [17, 19–22]. In a dog model of cerebral ischemia, neocortical and hippocampal pyramidal neurons, and cerebellar cortical Purkinje cells are more likely degenerated than other parts of brain [23]. Principal cells (neurons) in the somatosensory cortex are pyramidal cells (neurons) [19, 24]. It has been reported that, in the somatosensory cortex, pyramidal cells in layer III and pyramidal cells in the upper part of layer VI are much more sensitive to ischemia than the other cortical neurons [19]. Somatosensory inputs control complex senses and complex movements, therefore, the somatosensory cortex is crucial in neural rehabilitation in patients with brain lesions [17, 19, 24, 25].

Various factors contribute to neuronal cell damage after ischemic insults by several mechanisms, such as neuroinflammation, oxidative stress, etc. [16]. It is well known that inflammatory process in the central nervous system (CNS) leads to neuronal cell death following neurodegenerative diseases, including stroke, Parkinson’s disease, Alzheimer’s disease, and multiple sclerosis [26]. Inflammatory response in the CNS is mediated by activated microglia. Microglial activation amplifies inflammation-related neuronal injury in neurodegenerative diseases [26]. Some studies have focused on how HFD affects neural damage or death after ischemic insults [12, 13]. Also, we recently reported that HDF-induced obesity exacerbated neuronal injury in the hippocampus and striatum following transient forebrain ischemia (TFI) in gerbils [1, 11].

The aim of this study was to compare microgliosis and neuronal damage/death in the somatosensory cortex of HFD-fed gerbils with those of ND-fed gerbils after TFI. Gerbil are well used to investigate mechanisms of selective neuronal death following TFI [27–29].

## Materials and methods

### Experimental animals

Male gerbils were used at 6 months of age (body weight, 72–78 g). The gerbils were housed under a 12-h light/12-h dark cycle with constant temperature (22–23 °C) and relative humidity (55–60%). They were allowed free access to water and food (ND and HFD). All procedures in this study were in accordance with the guidelines, which are in compliance with the current international laws and policies from the “Guide for the Care and Use of Laboratory Animals” (The National Academies Press, 8th Ed., 2011). The experimental protocol of this study was approved (approval no., KW-200113-1) by “the Institutional Animal Care and Use Committee” at Kangwon National University (Chuncheon, Kangwon, Republic of Korea).

### Diet profile, and measurements of body weight, glucose level and lipid

All the gerbils were allowed free access to ND and HFD for 12 weeks. Rodent diet consisted of different fat concentrations as follows: ND (D12450B, 10% kcal % fat, 20% kcal % protein, 70% kcal % carbohydrate, Research Diets, NJ, USA) or HFD (D12492, 60% kcal % fat, 20% kcal % protein, 20% kcal % carbohydrate, Research Diets).

Body weight, blood glucose and serum lipid were measured at 12 weeks after ND or HFD according to our previously published method [30]. Briefly, the gerbils were deeply anesthetized by intraperitoneal injection of pentobarbital sodium (60 mg/kg) (JW Pharmaceutical, Seoul, Korea). Blood glucose level was analyzed through a blood glucose monitor (Ascensia Elite XL Blood Glucose Meter, Bayer, Toronto, ON, Canada) after collecting blood samples from each animal by orbital puncture. For measurement of total cholesterol and triglyceride level in the serum, serum was separated from the blood after centrifugation at 13,000 g for 30 min at 4 °C (centrifuge 5424R, Eppendorf, Hamburg, Germany), and analyzed using a dry chemistry analyzer (FUJI DRI-CHEM NX500; Fujifilm, Japan).

### Experimental groups

Gerbils (total *n* = 72) were randomly assigned to 4 groups. 1) ND/sham group: 15 gerbils (*n* = 5 at each time) were fed ND and received sham TFI; 2) ND/TFI group: 21 gerbils (*n* = 7 at each time) were fed ND and received TFI; 3) HFD/sham group: 15 gerbils (*n* = 5 at each time) were fed HFD and sham TFI; 4) HFD/TFI group: 21 gerbils were fed HFD and received TFI.

The gerbils in each group were sacrificed at 1 day, 2 days and 5 days, respectively, after TFI.

### Induction of TFI

TFI in the gerbil was induced according to our published method [1]. In short, the gerbils were anesthetized with a mixture of isoflurane (2.5%) in oxygen (34%) and nitrous oxide (66%). The occlusion of both common carotid arteries was done for 5 min, and reperfusion (restoration of blood flow) was done after 5-min TFI. The reperfusion was directly observed in the central artery in the retina with an ophthalmoscope (HEINE K180) (Heine Optotechnik, Herrsching, Germany). Body (rectal) temperature was controlled at normothermia (37 ± 0.5 °C). The sham TFI operation was done as follows. The surgical procedure was carried out except the occlusion of the common carotid arteries.

### Spontaneous motor activity (SMA)

We measured hyperactivity induced by TFI as ischemia-induced SMA at 1 day post-ischemia according to previous studies [11, 31]. In brief, the gerbils (*n* = 15 in each sham group, *n* = 21 in each ischemia group) were individually placed in a Plexiglas cage (25 cm × 20 cm × 12 cm) in a sound-attenuating chamber (Kinder Scientific, Poway, CA, USA). The cage was fitted with two parallel horizontal infrared beams that were located 2 cm from the floor. Movement was detected by interruption of an array of 32 infrared beams that was produced by photocells. Locomotor activity was recorded with Photobeam Activity System-Home Cage (San Diego Instruments, San Diego, CA, USA) during 60 min, simultaneously, and data for the locomotor activity were acquired by an AMB analyzer (IPC Instruments, Berks, UK). The data were evaluated in terms of entire distance (meters) that was traveled for 60 min of the test period. In this experiment, we did not expose all the gerbils to the open field prior to TFI.

### Preparation of histological sections

As described previously [1], all the gerbils were anesthetized with pentobarbital sodium (60 mg/kg, i.p.) at designated times after TFI. Their brains were rinsed with saline and fixed with solution of 4% paraformaldehyde via the ascending aorta. The fixed brains were removed and more fixed in the same fixative for 4 h. To make 30-μm coronal sections, the brains were infiltrated with solution of 30% sucrose and sectioned in a cryostat (Leica, Wetzlar, Germany).

### Immunohistochemistry

Immunohistochemistry was performed to examine (1) microglia activation with ionized calcium-binding adaptor molecule 1 (Iba-1, a marker for microglia) and (2) neuronal damage with neuron-specific soluble nuclear antigen (NeuN, a marker for neurons), as primary antibody. Immunohistochemistry with each antibody was done according to our published procedure [32]. In brief, the sections were incubated in each solution of primary antibody: rabbit anti-Iba-1 (1:1000, Wako Chemicals USA, Bermuda, VA, USA) and mouse anti-NeuN (1:1000, Chemicon, Temecula, CA, USA). These incubated sections were exposed to solution of biotinylated horse goat anti-rabbit and anti-mouse IgG (1:250, Vector Laboratories Inc., Burlingame, CA, USA), and streptavidin peroxidase complex (1:250, Vector, Burlingame, CA, USA). Finally, these reacted sections were visualized by using solution of 3,3′-diaminobenzidine (Sigma-Aldrich, St. Louis, MO, USA).

In order to establish the specificity of each immunostaining, negative control test was done with preimmune serum instead of each primary antibody. Each test did not show any immunoreactivity in the observed sections (data not shown).

### Fluoro-jade B (F-J B) histofluorescence staining

To investigate cell death/loss in the hippocampus after TFI, F-J B (Histochem, Jefferson, AR, USA) histofluorescence staining was done according to a published procedure [1]. In brief, the sections were immersed in solution of 1% sodium hydroxide, transferred to solution of 0.06% potassium permanganate and reacted with solution of 0.0004% F-J B on a slide warmer (about 50 °C).

### Data analysis

The immunoreactivity of Iba-1+ structure was quantitatively analyzed according to our published method [33]. In brief, digital image of Iba-1+ structure was taken like above-mentioned method. Iba-1+ image was calibrated into an array of 512 × 512 pixels. Immunoreactivity of Iba-1+ structure was evaluated as relative optical density (ROD), which was on the basis of an optical density (OD). OD was obtained after the transformation of the mean gray level by using a formula: OD = log (256/mean gray level). After the background density was subtracted, the ratio of the OD was calibrated with Adobe Photoshop 8.0 and analyzed as a percent, with sham-operated group designated as 100%, with NIH Image 1.59.

Neuronal damage and death/loss was quantitatively analyzed by NeuN immunohistochemistry and F-J B histofluorescence staining, respectively as follows. Six sections per gerbil were selected, and NeuN-immunoreactive (NeuN+) and F-J B-positive (F-J B+) cells were counted as previously described [34]. In short, digital images of NeuN+ and F-J B+ cells were obtained with light microscope (BX53) (Olympus, Tokyo, Japan) with blue (450–490 nm) excitation light, respectively. The images of the cells were captured in a 400 × 400 μm2 at the gerbil somatosensory cortex. Each cell count was done by averaging the total numbers by using image analyzing system (software: Optimas 6.5) (CyberMetrics, Scottsdale, AZ, USA).

### Statistical analysis

All statistical data were performed with GraphPad Prism (version 5.0; GraphPad Software, La Jolla, CA, USA) and expressed as means ± S.E.M. The significance of differences between the groups was assessed using two-way analysis of variance followed by post hoc Bonferroni’s multiple comparison. Differences were considered significant at *P* < 0.05.

## Results

### Characteristics of obesity

Body weight, blood glucose and serum lipid level were measured to examine whether HFD induced obesity in gerbils.

#### Changes in body weight

Body weight of the gerbils of the HFD group was significantly heavier (120.1 ± 2.2 g) than that in the ND group (79.8 ± 1.9) at 12 weeks after the feeding (Table 1).

**Table 1:** Changes in body weight, blood glucose and serum lipid in the ND-fed group and HFD-fed group

Parameters	ND (*n* = 36)	HFD (*n* = 36)
Body weight (g)	79.8 ± 1.9	120.1 ± 2.2*
Glucose (mg/dL)	106.5 ± 6.9	182.1 ± 4.8*
Triglyceride (mg/dL)	87.5 ± 7.3	167.3 ± 6.1*
Total cholesterol (mg/dL)	98.5 ± 5.6	183.9 ± 5.9*

Data are expressed as the mean ± S.E.M. *ND* normal diet; *HFD* high-fat diet. * *P* < 0.05 vs. ND-fed group

#### Changes in blood glucose

Blood glucose level in the HFD group were significantly increased (182.1 ± 4.8 mg/dL) at 12 weeks after the feeding compared to those in the ND group (106.5 ± 6.9 mg/dL) (Table 1).

#### Changes in serum lipid in gerbils

Serum triglyceride (167.3 ± 6.1 mg/dL) and total cholesterol (183.9 ± 5.9 mg/dL) levels in the HFD group were significantly increased at 12 weeks after the feeding compared to those in the ND group (87.5 ± 7.3 mg/dL and 98.5 ± 5.6 mg/dL, respectively) (Table 1).

### SMA

**Fig. 1:**
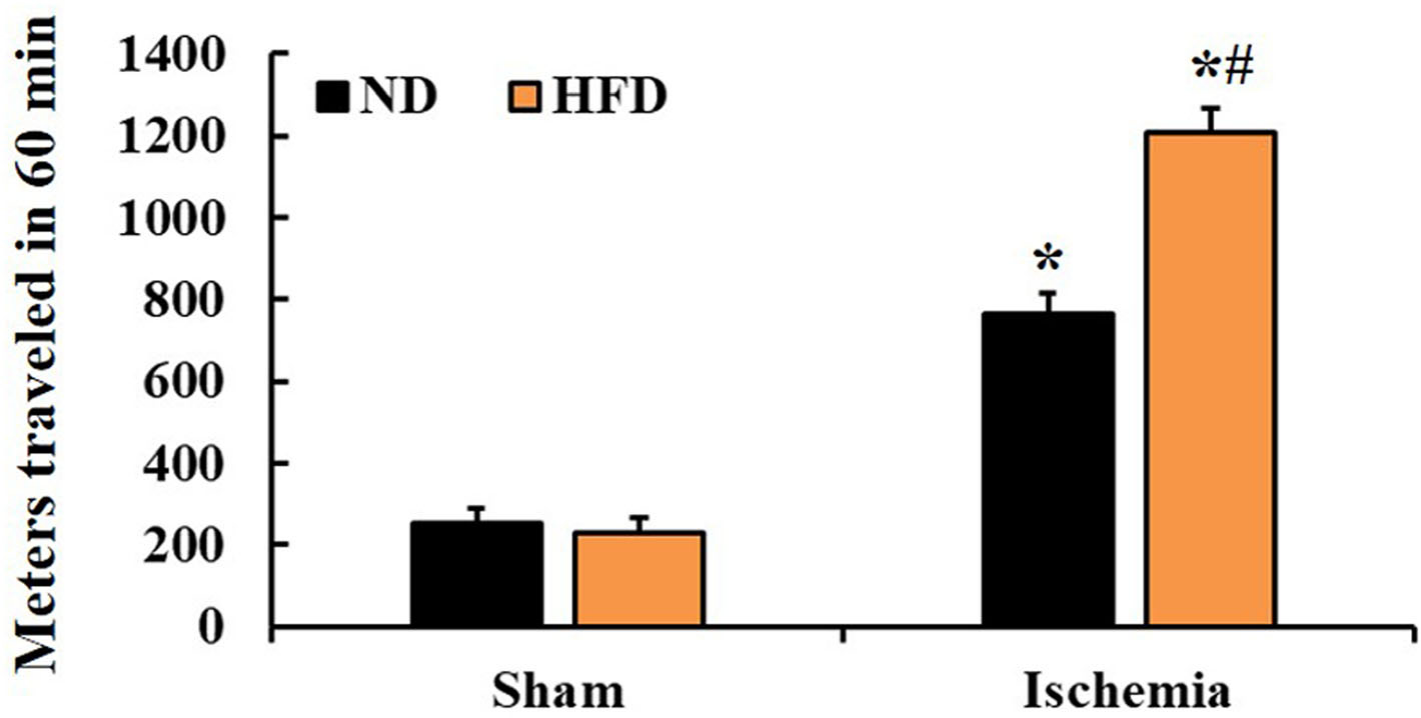
SMA of gerbils in the ND/sham, ND/TFI, HFD/sham, and HFD/TFI groups at 1 day post-TFI. SMA is evaluated by measuring entire distance (meters) traveled by gerbils (*n* = 15 in each sham group, *n* = 21 in each TFI group; ∗*P* < 0.05 vs. each sham group, #*P* < 0.05 vs. ND/TFI group). The bars indicate the means ± SEM

To examine ischemia-induced hyperactivity, SMA was measured by the total movement distance and evaluated at 1 day post-TFI. SMA in the HFD/sham group was similar to that in the ND/sham and (Fig. 1). In the ND/TFI group, SMA was significantly increased (about 202.7% of the sham group) at 1 day post-TFI compared to that in the ND/sham group, and, in the HFD/TFI group, SMA was significantly higher (about 174.3% of the ND/TFI group) than that in the ND/TFI group (Fig. 1). This result showed that TFI under HFD resulted in severer hyperactivity than that after TFI under ND.

### Microgliosis

**Fig. 2:**
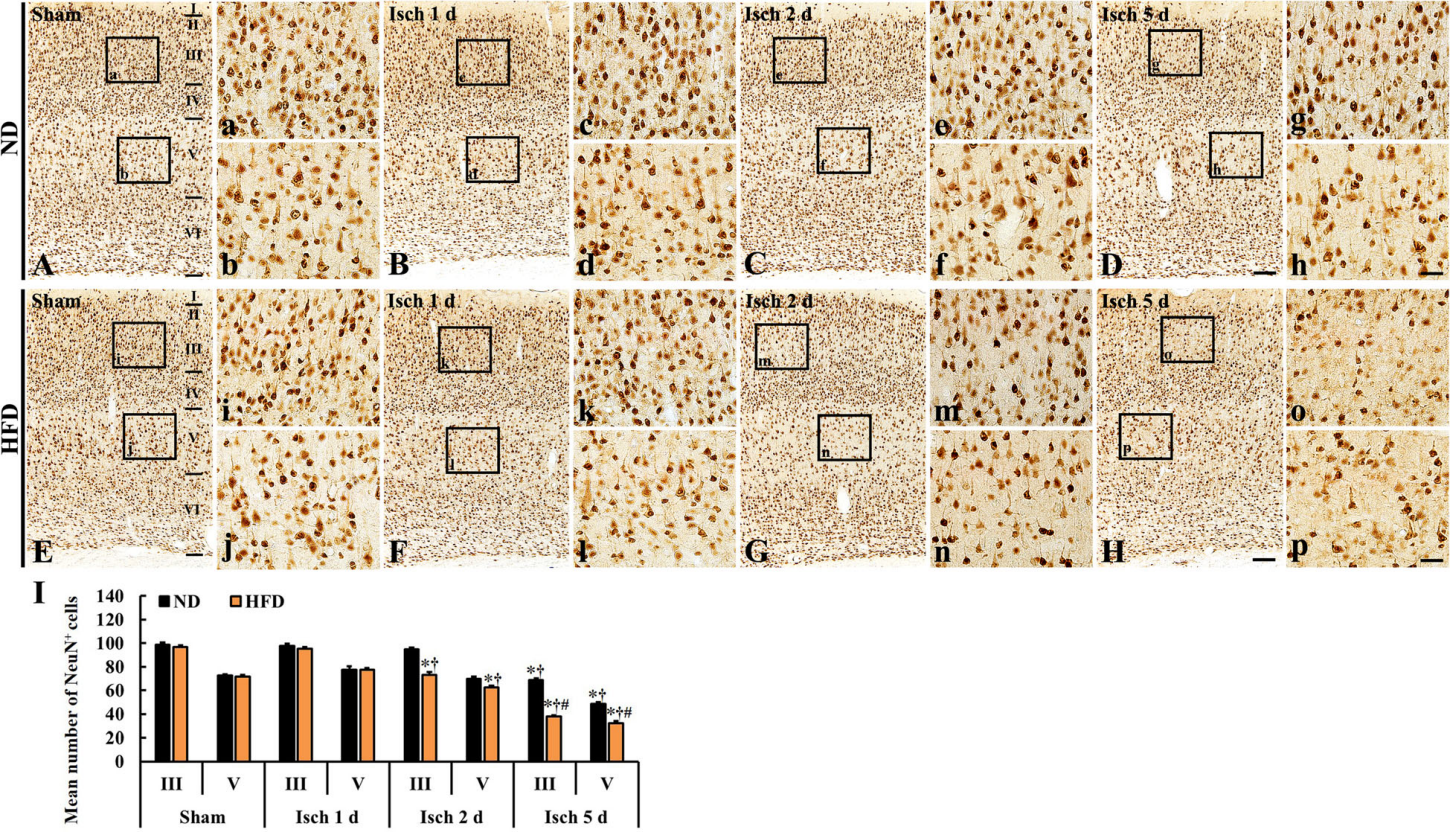
Iba-1 immunohistochemistry in the somatosensory cortex of the ND/sham (**A**), ND/TFI (**B-D**), HFD/sham (**E**), and HFD/TFI (**F-H**) groups at 1 day, 2 days, and 5 days after TFI. Microgliosis (hypertrophied Iba-1 immunoreactive cells and increased Iba-1 immunoreactivity) in the ND/TFI group is apparent at 5 days after TFI, however, microgliosis in the HFD/TFI group begins from 1 day after TFI and aggravates with time. Scale bar = 200 μm (**A–H**), 30 μm (**a–p**). **I** ROD of Iba-1 immunoreactive microglia in layer III and V (*n* = 5 in each sham group, *n* = 7 in each TFI group; ∗*P* < 0.05 vs. each sham group, **†***P* < 0.05 vs. pre-time point group, and #*P* < 0.05 vs. ND/TFI group). The bars indicate the means ± SEM

In the present study, we examined TIF-induced microgliosis, which means that microglial cells are activated, in the somatosensory cortex using Iba-1 immunohistochemistry (Fig. 2). Iba-1 immunoreactive microglia showed a resting form in both ND/sham (Fig. 2 A, a, b) and HFD/sham groups (Fig. 2 E, i, j).

In the ND/TFI and HFD/TFI groups, Iba-1 immunoreactive microglia were activated in layer III and V, in which pyramidal cells (principal neurons in the somatosensory cortex) from 1 day post-TFI (Fig. 2). ROD of Iba-1 immunoreactive microglia in the ND/TFI group was slightly increased at 1 day post-TFI (Fig. 2 B, c, d); at this point in time, however, ROD of Iba-1 immunoreactive microglia in the HFD/TFI group was significantly higher (about 21.5% in layer III and 35.4% in layer V) than that in the in the ND/TFI group (Fig. 2 F, k, l, I). At 2 days post-TFI, ROD in the HFD/TFI group (Fig. 2 G, m, n, I) was more increased (14.2% in layer III and 22.4% in layer V) than that ND/TFI group (Fig. 2 C, e, f, I). ROD at 5 days post-TFI was more increased in both groups (Fig. 2 D, g, h, H, o, p) than that at 2 days post-TFI, but the ROD in the HFD/TFI group was slightly higher than that in the ND/TFI group (Fig. 2 I). This result showed that TFI following HFD resulted in earlier and higher microglial activation in the somatosensory cortex than that after TFI under ND.

### Neuronal damage and death (loss)

#### NeuN immunoreactive cells

**Fig. 3:**
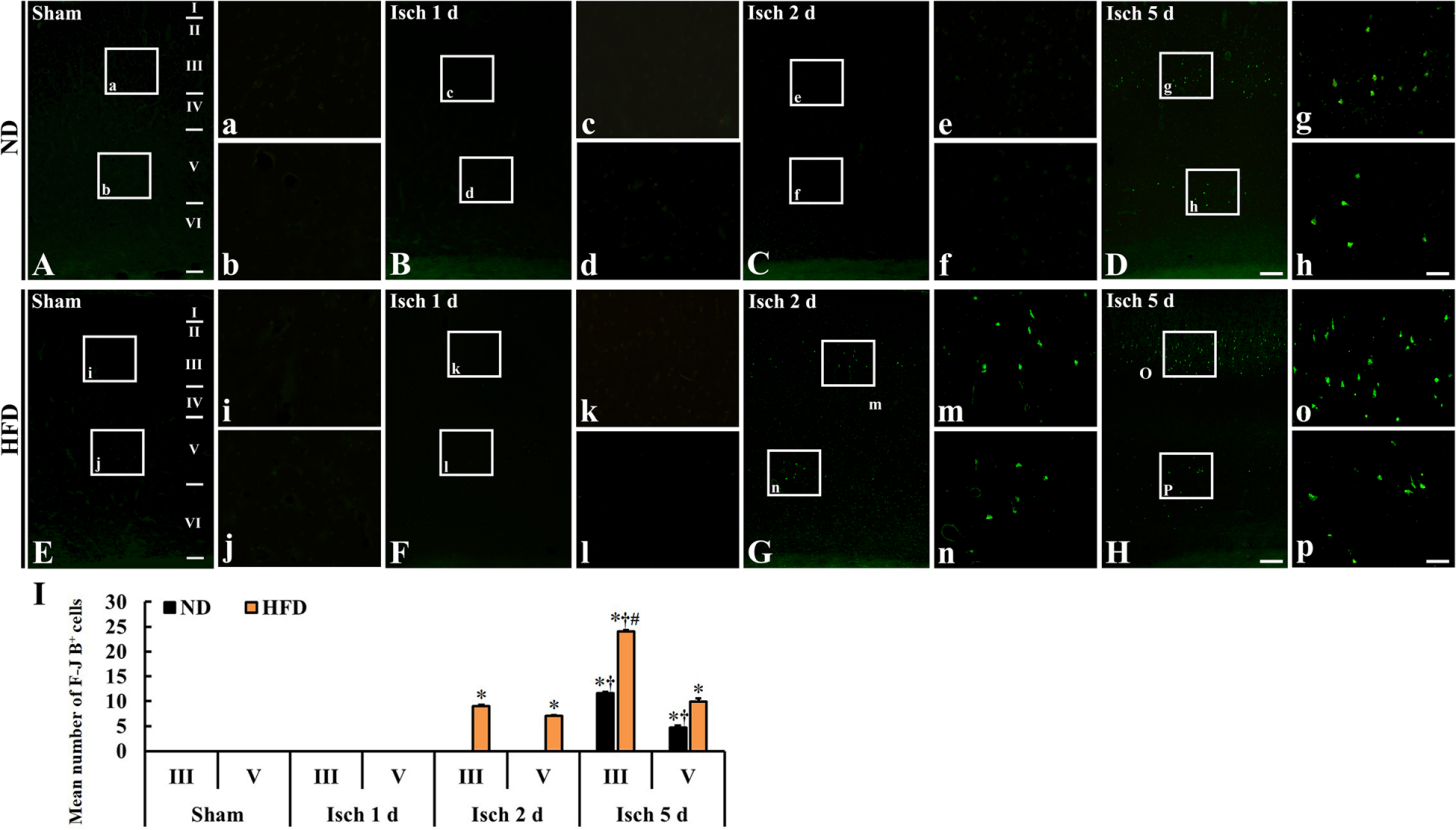
NeuN immunoreactive cells in the somatosensory cortex of the ND/sham (**A**), ND/TFI (**B-D**), HFD/sham (**E**), and HFD/TFI (**F-H**) groups at 1 day, 2 days, and 5 days after TFI. NeuN immunoreactive neurons in layer III and V of the HFD/TFI group are decreased from 2 days after TFI, but, in the ND/TFI group, NeuN immunoreactive neurons are decreased at 5 days after TFI. Note that numbers of NeuN immunoreactive neurons are significantly lower in the HFD/TFI group than ND/TFI group at 5 days after TFI. Scale bar = 200 μm (**A–H**), 30 μm (**a–p**). **I** Men number of NeuN immunoreactive cells in layer III and V (*n* = 5 in each sham group, *n* = 7 in each TFI group; ∗*P* < 0.05 vs. each sham group, **†***P* < 0.05 vs. pre-time point group, and #*P* < 0.05 vs. ND/TFI group). The bars indicate the means ± SEM

In this study, we examined TFI-induced neuronal damage by observing NeuN immunoreactive cells, which are intact neurons that contain neuronal nuclei, in layers III and V because changes in neurons as well as microglial cells were generally shown in layers III and V (Figs. 2 and 3). In both ND/sham and HFD/sham groups, neurons in the gerbil somatosensory cortex were well stained with NeuN (Fig. 3 A, a, b, E, i, j), and numbers of NeuN immunoreactive neurons were not different between each group (Fig. 3 I).

One day after TFI, NeuN immunoreactive neurons in layers III and V were not significantly altered in both ND/TFI (Fig. 3 B, c, d) and HFD/TFI (Fig. 3 F, k, i) groups compared those in both sham groups (Fig. 3 I). At 2 days post-TFI, the number of NeuN immunoreactive neurons in the ND/TFI group was not changed (Fig. 3 C, e, f), however, the number of NeuN immunoreactive neurons in the HFD/TFI group was significantly decreased (about 23.9% in layer III and 8.9% in layer V) compared to that in the sham group (Fig. 3 G, m, n, I). At 5 days after TFI, the number of NeuN immunoreactive neurons in the ND/TFI group was significantly decreased (about 30.1% in layer III and 24.4% in layer V) compared with that in the sham group (Fig. 3 D, g, h, I). At this point in time, the number of NeuN immunoreactive neurons in the HFD/TFI group was dramatically reduced (about 31.5% in layer III and 16.5% in layer V) compared with that in the ND/TFI group (Fig. 3 H, o, p, I). This result showed that TFI under HFD led to a faster and greater reduction in the number of neurons in the somatosensory cortex than that after TFI under ND.

#### F-J B positive cells

**Fig. 4:**
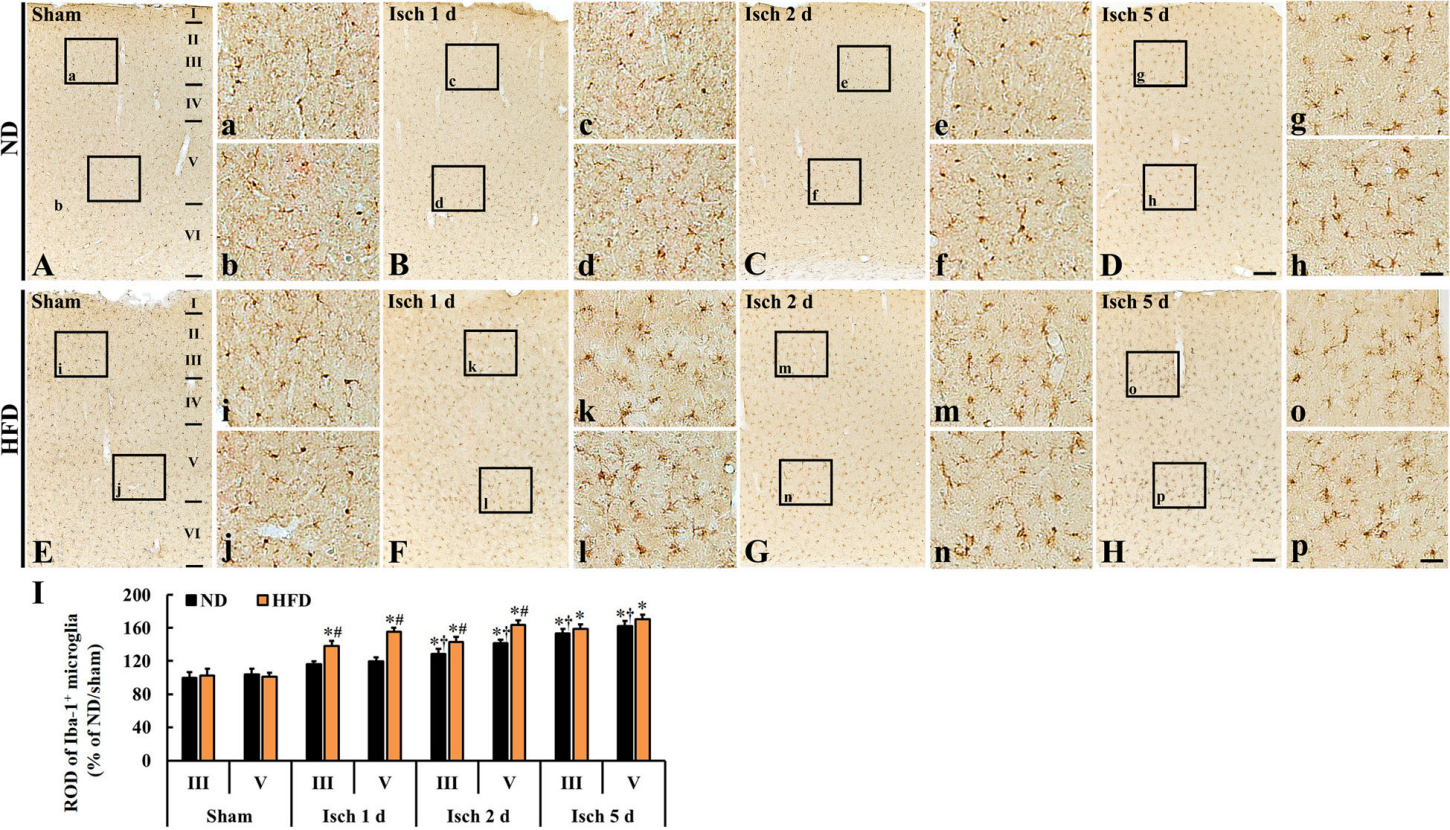
F-J B positive cells in the somatosensory cortex of the ND/sham (**A**), ND/TFI (**B-D**), HFD/sham (**E**), and HFD/TFI (**F-H**) groups at 1 day, 2 days, and 5 days after TFI. F-J B positive cells in the ND/TFI group appear in layer III and V at 5 days after TFI, however, in the HFD/TFI group, F-J B positive neurons appear from 2 days after TFI. Note that numbers of F-J B positive cells are higher in the HFD/TFI group than ND/TFI group at 5 days after TFI. Scale bar = 200 μm (**A–H**), 30 μm (**a–p**). **I** Mean number of F-J B positive cells in layer III and V (*n* = 5 in each sham group, *n* = 7 in each TFI group; ∗*P* < 0.05 vs. each sham group, **†***P* < 0.05 vs. pre-time point group, and #*P* < 0.05 vs. ND/TFI group). The bars indicate the means ± SEM

In this study, TFI-induced neuronal death (loss) was examined by observing F-J B positive cells, which are degenerating or dead cells, in layer III and V (Fig. 4). In both ND/sham and HFD/sham groups, no F-J B positive cells were observed in any layers of the somatosensory cortex (Fig. 4 A, a, b, E, I, j).

On day 1 after TFI, F-J B positive neurons were not shown at 1 day post-TFI in both ND/TFI (Fig. 4 B, c, d) and HFD/TFI (Fig. 4 F, k, l) groups. At 2 days post-TFI, F-J B positive cells were not detected in the ND/TFI group (Fig. 4 C, e and f), however, many F-J B positive cells were shown in layer III and V in the HFD/TFI group (Fig. 4 G, m, n, I). At 5 days post-TFI, F-J B positive cells were found in layer III and V in the ND/TFI group (Fig. 4 D, g, h), showing that the numbers were similar to those in the ND/TFI group at 2 days post-TFI (Fig. 4h). At this point in time, the number of F-J B positive neurons in the HFD/TFI group was significantly increased in layer III (about 107.4%) and V (about 109.0%) compared with that in the ND/TFI group (Fig. 4 H, o, p, I). This finding indicates that HFD accelerates and exacerbates neuronal death in the somatosensory cortex after TFI.

## Discussion

Our findings indicate that HFD can evoke earlier microgliosis and more detrimental neuronal death/loss in the somatosensory cortex after transient ischemia than ND evokes.

In the present study, body weight of the gerbils fed HFD for 12 weeks was significantly increased compared with the gerbils fed ND. We recently reported that the level of blood glucose, triglyceride, total cholesterol, and low-density lipoprotein cholesterol levels were significantly increased in this gerbil model of HFD [1]. In addition, it has been reported that, in gerbils fed HFD for 8 weeks, body weight, level of blood glucose, triglyceride and total cholesterol are significantly increased compared with gerbils fed ND [35]. The negative effect of obesity in gerbils was observed after 4–6 months of HFD feeding [36]. Above-mentioned authors have reported that ischemia-reperfusion injury exacerbates neuronal damage in the striatum [1] and septum [35] of gerbils fed HFD. In the case of rats, HFD feeding for 3 months worsens the outcome of neuronal damage after endothelin-induced ischemia [14]. Taken together, we suggest that HFD feeding for long time can induce severe neuroinflammation and neuronal damage/death in brains following ischemic insults.

Microglia are resident macrophages and distributed throughout brain parenchyma. Microglia are generally activated in early response to various pathological stimuli, including trauma, inflammation, and degeneration [37]. In addition, it has been reported that microglia in the hippocampus is closely related to neuronal degeneration following various CNS diseases [38], type 2 diabetes [39] and aging [40]. Namely, microglia exert cytotoxic function by releasing reactive oxygen species (ROS), nitric oxide and/or inflammatory cytokines, which trigger neuronal damage [37, 41, 42]. In this study, we used Iba-1, as a marker for microglia, that is expressed in microglia in the brain and used [37], and found that ROD of Iba-1-immunoreactive microglia was gradually increased in layer III and V of the gerbil somatosensory cortex in both ND/TFI and HFD/TFI groups, but the number of Iba-1-immunoreactive microglia in the HFD/TFI group were accelerated and exacerbated compared with those in the ND/TFI group. This finding indicates that microgliosis is severer in brains of animals fed HFD than that in ones fed ND. In previous studies, Iba-1 immunoreactive cells appeared rapidly (in a few hours after ischemia) in peri-ischemic area following transient focal cerebral ischemia in rats, suggesting that microgliosis (microglial activation) might reflect the extent of severity of focal ischemic injury [37, 43].

It has been reported that changes in function and morphology of microglia are closely correlated with the development of delayed neuronal death after transient forebrain ischemia in gerbils [19, 20]. Namely, activated microglia release a variety of cytotoxic agents that lead to neuronal damage/death in the hippocampus [19, 44]. In addition, some researchers have suggested that activation of microglia in the brain indicates a hallmark of brain pathology [26, 45]. In addition, we have reported that HFD-induced aggressive neuronal death in the dorsolateral striatum is closely related to neuroinflammation (microglial activation and increased tumor necrosis factor-alpha and interleukin-1beta expressions) after 5 mins of TFI, and this increase in inflammation is attenuated by mTOR inhibitor rapamycin [1]. These studies lead to conclusion that rapid and severe microglia activation following ischemic insults is related to acceleration of neuronal damage or death in experimental animals fed HFD. Based on this idea, we compared neuronal damage or death in the somatosensory cortex between the ND/TFI and HFD/TFI gerbils by using NeuN immunohistochemistry and F-J B histofluorescence staining. We found that TFI-induced neuronal death/loss in layer III and V of the somatosensory cortex in the ND-fed gerbils was found only at 5 days post-TFI, but, in the HFD-fed gerbils, neuronal death/loss occurred from 2 days post-TFI and exacerbated at 5 days post-TFI. It is known that pyramidal cells in layer III and small pyramidal cells in the upper part of layer VI of the somatosensory cortex are much more sensitive to ischemia than the other cortical neurons [19]. Somatosensory inputs control complex senses and movements [17, 19]. For instance, proprioceptive and tactile inputs play a role for balancing the position of the body in space and for the refinement of motor control [24, 25]. In this regard, somatosensory stimulation may be crucial in neural rehabilitation by influencing motor function in patients with brain lesions, including ischemic stroke [17, 25].

As described above, in this study, the loss of NeuN-immunoreactive neurons in the somatosensory cortex of the HFD/TFI group was significantly rapid compared with that in the ND/TFI group. NeuN protein is located in the nucleus, so immunohistochemical analysis of anti-NeuN antibody has been exclusively applied to nervous tissue to detect neurons [46]. The expression of NeuN is associated with neuronal differentiation and plays a permanent regulator of general presentation of neuronal phenotype throughout whole cell life. Therefore, NeuN has been widely used as a marker to identify histopathologic diagnosis (34). The expression of NeuN protein can be affected by nervous system injury. For example, NeuN decreases in damaged or dying pyramidal neurons in the hippocampus, and NeuN immunoreactivity is reduced in hypoxia and brain injury [46–48]. It is reported that loss of NeuN immunoreactivity is explained by neuronal damage in damaged brain areas (34). In the case of brain ischemia, numbers of NeuN immunoreactive neurons are more decreased in the somatosensory cortex after a longer ischemia in gerbils [19].

F-J B is used as a marker for study on neuronal degeneration after ischemic injury because it has a good affinity for entirely degenerating neurons (cell bodies, dendrites, axons and axon terminals) [49]. Obvious F-J B positive neurons are sown in the hippocampus of the rat injured by mid and moderate level of traumatic brain injury [50]. In the case of ischemia, Cao et al. (2015) reported that greater numbers of F-J B positive neurons in the brain are related to degree of ischemic injury in rat models of mild and moderate focal cerebral ischemia induced by middle cerebral artery occlusion [51]. In our current study, F-J B positive pyramidal neurons in the gerbil somatosensory cortex are found earlier and more in number in the HFD/TFI than those in the ND/TFI group. These results indicate that HFD accelerates ischemic neuronal death in the somatosensory cortex following TFI.

Besides, it has been reported that obesity-induced exacerbation of ischemic brain damage is associated with secretion of proinflammatory mediators [7, 51, 52]. In this regard, we recently reported that HFD-induced obesity in gerbils increased pro-inflammatory cytokines and mTOR activation, and elicited neuronal death in the striatum after TFI [1]. In addition, Fifield et al. (2019) recently reported that obesity developed chronic low-grade inflammation that increased the release of inflammatory mediators and immune cell infiltration into the brain following transient brain ischemia [2]. Therefore, we have to examine changes in pro-inflammatory cytokine expressions in ND/TFI and HFD/TFI groups, although we did not compare changes of pro-inflammatory cytokines in the somatosensory cortex between the ND/TFI and HFD/TFI groups.

In conclusion, degrees of microgliosis and neuronal damage/death in the somatosensory cortex following TFI in gerbils fed HFD were exacerbated compared with that in gerbils fed ND. These results indicate that HFD accelerates and exacerbates neuronal injury in the somatosensory cortex after transient brain ischemic insults via accelerating severer microgliosis.

## Data Availability

All data produced and analyzed in the present study are included in this published paper.
